# Effects of different fencing regimes on community structure of degraded desert grasslands on Mu Us desert, China

**DOI:** 10.1002/ece3.4958

**Published:** 2019-02-28

**Authors:** Jiankang Liu, Zhen Bian, Kebin Zhang, Bilal Ahmad, Alamgir Khan

**Affiliations:** ^1^ School of Soil and Water Conservation Beijing Forestry University Beijing China; ^2^ Key Laboratory of State Forestry Administration on Soil and Water Conservation Beijing Forestry University Beijing China; ^3^ School of Water Conservancy and Environment University of Jinan Jinan China

**Keywords:** aboveground biomass, community structure, degraded desert grassland, fencing, species diversity

## Abstract

Grazing is one of the major anthropogenic driving factors influencing community structure and ecological function of grasslands. Fencing has been proved to be one of the main measures for rehabilitating degraded grasslands in northwestern China. However, data from combined empirical studies on the effects of different management regimes in desert grasslands are lacking. So we selected long‐term fencing (fenced since 1991), mid‐term fencing and seasonal fencing (fenced since 2002), and adjacent free‐grazing grasslands to investigate vegetation and soil properties on southwest Mu Us desert. Our results showed that fencing increased plant cover, height, aboveground biomass (AGB) of different plant life‐form groups, Shannon–Wiener diversity index, Evenness index, Simpson index, total soil nitrogen, total soil phosphorus, and soil organic matter, but decreased plant density, species richness, Richness index, soil bulk density, water content, and pH. However, 22–24 years of long‐term complete fencing might cause redegradation of vegetation and soil nutrients, characterized by the reduction of some vegetation properties, biodiversity, total AGB, and some soil properties. Seasonal fencing with 11–13 year was more beneficial to vegetation restoration than that with completely fencing measures. Our study suggests that appropriate artificial disturbances, such as seasonal fencing (winter grazing and summer fencing), should be used after long‐term fencing in order to maintain grassland productivity and biodiversity. These findings will help to provide theoretical support for vegetation restoration and sustainable management in grassland under grazing prohibition at Mu Us desert.

## 1 INTRODUCTION

Grazing, which has been widely considered as the main anthropogenic driver of vegetation degradation and environment ecosystem deterioration (Akiyama & Kawamura, 2007; Hobbs & Huenneke, 1992; Nedessa, Ali, & Nyborg, 2005), is one of the most direct and essential factors for promoting community succession, changing community structure and ecological function in arid and semiarid areas (Akiyama & Kawamura, 2007; Wu, Du, Liu, & Thirgood, 2009). The climate in these areas is generally characterized by strong sunlight, high temperature, high evapotranspiration, and little precipitation (Ren, Jia, Wan, Han, & Chen, 2011), inducing a fragile ecological ecosystem that is sensitive to human activities and climate change (Zuo et al., 2014). Moderate grazing can maintain community diversity according to the “intermediate disturbance hypothesis” (Catford et al., 2012; Connell, 1979). However, overgrazing leads to the excess output of energy and nutrient from plant–soil ecosystems to herbivores which results in degradation of grasslands (Chartier, Rostagno, & Pazos, 2011). Overgrazing pattern aimed to get higher economic income may not only reduce vegetation cover, aboveground productivity, species diversity, and change the distribution of community spaces, but also exacerbate soil erosion and induce soil degradation, or even enhance desertification (Connell, 1979; Mekuria & Aynekulu, 2013; Nedessa et al., 2005; Wu et al., 2009). Presently, most of grasslands in northwest China show different degrees of degradation due to overuse (Wu & Loucks, 1992). How to restore degraded grasslands has become an urgent problem to be solved in recent decades.

Improving grazing management regimes is considered a main strategy to reestablish degraded grasslands (Lal, 2004; Mekuria & Aynekulu, 2013). Although a variety of biological and engineering practices have been carried out to restore the degraded grassland and protect undegraded grassland, exclosure is one of the most useful measures for restoring vegetation due to its low investment, extensiveness, simple implementation, and quick response (He, Zhao, Liu, Zhao, & Li, 2008; Liu, Zhang, Wang, & Yang, 2015). The effects of excluding livestock grazing on plant communities in arid and semiarid regions mainly depend on the duration of exclosure (Angassa & Oba, 2010) and some environmental factors, especially precipitation (Huxman et al., 2004; Miao, Guo, Xue, Wang, & Shen, 2015). Previous studies had indicated that fencing could significantly change species composition, improve soil physicochemical properties, and promote ecosystems resilience (Li, Cao, et al., 2017; Li, Zhang, et al., 2017; Schmiede, Donath, & Otte, 2009; Shang et al., 2013; Wu et al., 2009) through direct or indirect approaches (Socher, Prati, Boch, Müller, & Fischer, 2012) to restore degraded grassland ecosystem in a short time (Rooyen, Roux, Geldenhuys, Rooyen, & Merwe, 2014), and finally improve the growth and livelihood environment condition for humans (Gao et al., 2009; Shang, Ma, Long, & Ding, 2010). Shang et al. (2013) indicated 3 years of fencing could alter species composition and increase plant cover. Grassland with 6–8 years of fencing had higher aboveground vegetation productivity and cover, but lower plant density and species diversity compared with adjacent grazing grassland (Wu et al., 2009). Some studies showed that the 12 years of fencing significantly increased vegetation cover, height, Richness index, above‐ and belowground biomass (Zhu, Deng, Zhang, & Shangguan, 2016). However, because of lacking in human interference, the fierce competition of plants, caused by limited resources, may induce intense intra‐ and interspecific competition in exclosure grasslands (Angassa & Oba, 2010; Catford et al., 2012; Connell, 1979; Oba, Vetaas, & Stenseth, 2001). This may lead to a loss of biodiversity and ultimately cause redegradation of ecosystem after long‐term fencing (Shang et al., 2010; Su, Liu, Xu, Wang, & Li, 2015).

Recent studies focused on the effects of grazing bans/exclosure mostly concentrated on plant community characteristics (Deng, Zhang, & Shangguan, 2014; Li, Zhang, et al., 2017; Li, Cao, et al., 2017; Liu et al., 2015; Rooyen et al., 2014; Wal, Bardgett, Harrison, & Stien, 2004; Wu et al., 2009) and soil properties (Li, Zhang, et al., 2017; Li, Cao, et al., 2017; Mekuria & Aynekulu, 2013; Zhu et al., 2016; Zou et al., 2016). Plenty studies have focused on the evaluation of aboveground vegetation and soil based on different restoration time or management types (Su et al., 2015; Zhu et al., 2016). However, there is a lack of research on long‐term exclosure desert grasslands (e.g., >20 years).

Because of excessive grazing, local vegetation in Mu Us desert, northwest China, has been seriously degraded in late 20th century. A variety of restoration measures, including fencing, have been implemented to restore the local environment in study area. In order to (a) assess the effects of different grassland management regimes on plant community and soil, and (b) reveal the relationship between community composition, aboveground biomass (AGB), biodiversity, and soil properties, four sites with different fencing time or management regimes were selected in this research. The change observed can be used as indicators of exclosure effectiveness and provide a foundation for sustainable management and utilization of restoring other grasslands in arid and semiarid areas.

## 2 MATERIALS AND METHODS

### 2.1 Study site

**Figure 1:**
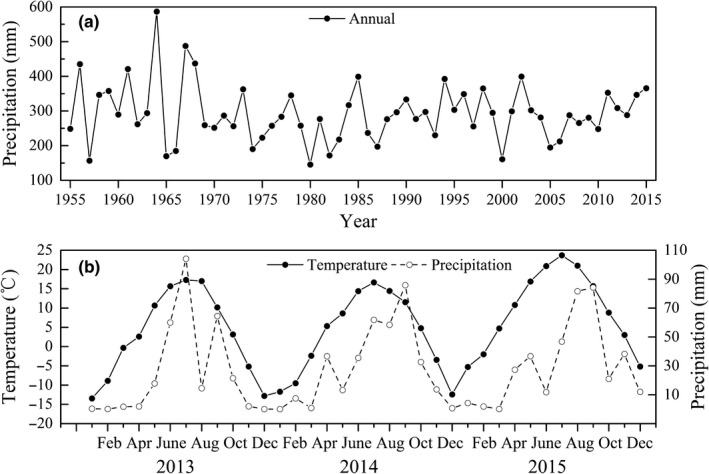
Variation of annual average precipitation from 1955 to 2015 (a) and monthly precipitation from 2013 to 2015 (b) in research area

Field experiment was conducted at the Liuyangpu artificial enclosed area, which is located in Yanchi County (37°04′N–38°10′N, E106°30′N–107°41′N, 1,295 m‐1,951 m a.s.l), Ningxia Hui Autonomous Region, northwest China. The study area lies in the southwest of Mu Us desert between arid and semiarid climatic zones. The terrain of Yanchi County is mainly for denudation plain and diminishes from south to north gradually. The climate is temperate continental climate, dry and windy. The average annual precipitation is about 285 mm (145.3 mm to 586.8 mm), but over eighty percent of precipitation occurs in the growing season from June to September (Figure 1). The mean annual air temperature is 8.1°C (−24.2°C to 34.9°C). The mean annual potential evaporation is 2,024 mm. The average frost‐free period is about 165 days. The plant growing stage is mainly between the ends of April to the end of October. The study area is dominated by shrub, subshrub, and tall perennial herbs communities, such as *Artemisia ordosica, Caragana korshinskii, Heteropappus altaicus, Salix psammophila, *and *Artemisia scoparia*. The meteorological data for this study area were provided by the meteorological station of Yanchi County.

### 2.2 Experimental design and field survey

We used spatiotemporal approach to monitor the effects of fencing. Four adjacent treatment sites: long‐term completely fencing grassland (LG), mid‐term completely fencing grassland (FG), mid‐term seasonal fencing grassland (SG), and continued free‐grazing grassland (CG), were selected in this study according to different management regime and fencing times. Each treatment site was about 20 ha (500 m × 400 m), and less than 10 meters between each plot. LG and FG sites were completely fenced since 1991 and 2002, respectively. SG site was fenced in 2002, however, grazed at the end of growing season (autumn and winter) with a medium livestock density of 0.75 *Ovis aries* per ha in this area (Liu, Xie, Dai, & Rao, 2008). CG site was with unlimited access for free traditional grazing with a moderate to severe grazing intensity of 1 sheep per ha (Liu et al., 2008). The pore size of fences in the study area was greater than 15 cm which could remove large livestock, while some small herbivores, such as *Lepus sinensis* and *Microtinae*, were able to pass through the fences and active in exclosure area. The main characteristics of each site are shown in Table 1. The four sampling sites were in one common pasture under similar livestock grazing intensity before fenced, which ensure a relatively homogeneous vegetation conditions. They were also in the same continuous flat area and adjacent to each other, ensuring a comparable edaphic conditions. This disturbance and geographical conditions effectively reduced the potential for environmental spatial heterogeneity and resulted in a relatively homogeneous soil conditions.

**Table 1:** Basic information of site characteristics

Field	Coordinate	Altitude (m)	Soil type	Slope orientation	Slope angle (°)	Interference conditions	Community types
LG	37°50′46.1″N 107°24′07.8″E	1,396	Sierozem	Southwest	1–3	No	*Salsola ruthenica*, *Artemisia scoparia*
FG	37°50′45.6″N 107°23′58.4″E	1,396	Sierozem	Southwest	1–3	No	*Artemisia ordosica*, *Heteropappus altaicus*
SG	37°50′46.3″N 107°23′48.3″E	1,394	Sierozem	Southwest	2–3	Grazing in autumn and winter	*Artemisia ordosica*, *Heteropappus altaicus*, *Salsola ruthenica*
CG	37°50′30.7″N 107°24′37.6″E	1,395	Sierozem	Southwest	2–4	Continued grazing	*Artemisia ordosica*, *Salsola ruthenica*

In each treatment site, five 30 m × 30 m plots were established randomly with minimum distance of 50 m. Each plot was laid more than 20 m inside the margin of fencing to avoid edge effects. In these plots, three 2 m × 2 m subplots, treated as parallel set, were randomly selected to represent the vegetation condition. In each 2 m × 2 m subplot, we performed a quantitative vegetation inventory, including plant species, density, coverage, height, and AGB. And the average plant indicators of the three sample subplots were used to represent the plant indicators of the 30 m × 30 m plot. All plants in each subplot were cut from the ground and then weighed after oven‐drying at 60°C for 48 hr to constant weight. All vegetation surveys were conducted in mid‐August from 2013 to 2015, when plant biomass reached its maximum height.

### 2.3 Soil sampling

We sampled soil at the same time of the vegetation surveys. In each 30 m × 30 m plot, five randomly selected soil samples at the depth of 0–30 cm were taken by bucket auger (7.5‐cm inner diameter). These samples then air‐dried and mixed into a single sample. In each site, five samples were collected. All samples were passed through a 0.15‐mm sieve for remove root, and the following nutrients were measured (Ma, Ding, & Li, 2016; Wu et al., 2009). Soil organic matter (SOM, g/kg) was analyzed by dichromate oxidation (Nelson & Sommers, 1982), total nitrogen (TN, g/kg) was used by Kjeldahl method (Bremner, 1996), and total phosphorus (TP, g/kg) was determined after digestion of soil with HClO_4_–H_2_SO_4 _(Parkinson & Allen, 1975). Soil bulk density (BD, g/cm^3^) was measured by the volumetric ring method (Wu, Liu, Zhang, Chen, & Hu, 2010). Soil water content (SWC, %) was measured by the oven‐dried method (Li, Zhang, et al., 2017; Li, Cao, et al., 2017). Soil pH was measured in a soil: water ratio aqueous extract of 1:5 (PHS‐3C pH acidometer, China). Each soil sample was measured with three replicates to ensure data accuracy.

### 2.4 Diversity index calculation

Plant species richness index (*R*), Shannon–Wiener diversity index (*H*), Simpson dominance index (*D*), and Evenness index (*E*) were used to illustrate biodiversity in this study. These indices were calculated as the following formulas (Ulanowicz, 2001):

Richness index (*R*):

(1) R=(S-1)/lnN

Shannon–Wiener index (*H*):

(2) $$H=-\sum_{i=1}^{S}\left(P_{i}\ln P_{i}\right)$$

Simpson dominance index (*D*):

(3) $$D=1/\sum_{I=1}^{S}\left(P_{i}\ln P_{i}\right)$$

Evenness index (*E*):

(4) E=H/lnS

where *S* is the total species number found in each quadrat, *N* is the summation of plant importance values in limited area, *P_i_* is the relative important value represented by *i*th species.

### 2.5 Data analysis

Plant community and soil indicators were analyzed to evaluate the effects of various management regimes on desert steppe in all survey years. Repeated measures analysis of variance (RANOVA) models were used to examine the effects of fencing regimes (FR), years (Y), and (FR × Y) on plant and soil properties, in which FR and SY were fixed factors. The Pearson correlation texts were conducted to analyze the relationship between community properties and soil physicochemical properties. Significant differences were evaluated at the level of *p* < 0.05 in the following sections. All statistical analyses were performed using EXCEL 2013 and SPSS 20.0 software (SPSS for Windows, Chicago, USA). All figures were accomplished by OriginPro 2015 (OriginLab Corporation 2015).

## 3 RESULTS

### 3.1 Community properties

ANOVAs indicated that fencing regimes had significant effects (*p* < 0.01) on community properties apart from H (Table 2). Our results showed that different patterns of fencing (LG, FG, and SG) significantly increased (*p* < 0.05) plant cover and height among 3 years, however, decreased (*p* < 0.05) plant density in 2013 and 2015 (Table 3). The plant cover in all survey years comply with the order of FG > SG > LG > CG, while plant density was in order of CG > SG > FG > LG. The plant cover in LG, FG, and SG sites increased by 1.60, 2.30, and 1.66 times at 2013, increased by 1.26, 1.42, and 1.40 times at 2014, while increased by 1.37, 1.95, and 1.66 times at 2015 compared with CG site, respectively. Plant height increased by 3.64, 3.67, and 3.42 times at 2013, increased by 1.62, 1.34, and 1.20 times at 2014, while increased by 1.41, 1.83, and 1.78 times at 2015 compared with CG site, respectively. Plant density yet decreased by 52.5, 47.7, and 36.6 percent at 2013, decreased by 19.2, 11.4, and 5.7 percent at 2014, while decreased by 54.3, 31.4, and 24.9 percent at 2015 compared with CG site, respectively. Plant cover in four survey sites and height in LG and CG sites at rainy years (2014 and 2015) were higher than normal precipitation year (2013) (Table 3).

**Table 2:** Repeated measures ANOVA results for the effects of fencing regimes (FR), years (Y), and there interaction (FR × Y) on cover, height, density, Shannon–Wiener diversity index (*H*), Evenness index (*E*), Simpson index (*D*), Richness index (*R*), aboveground biomass (AGB), species richness (SR), soil bulk density (BD), soil water content (SWC), total nitrogen (TN), total phosphorus (TP), soil organic matter (SOM), and pH

		Cover	Height	Density	*R*	*H*	*D*	*E*	AGB	SR	BD	SWC	TN	TP	SOM	pH
Y	*F*	64.2	35.15	2.552	2.844	1.26	9.105	3.136	224.5	2.571	7.932	172.5	0.435	5.437	2.788	8.25
	*p*	<0.001	<0.001	0.096	0.117	0.334	<0.01	0.099	<0.001	0.094	<0.05	<0.001	0.662	<0.05	0.121	0.011
FR	*F*	11.624	50.286	39.285	60.928	0.543	21.572	6.311	113.536	6.686	166.59	732.117	261.56	1,174.01	144.163	108.265
	*p*	<0.001	<0.001	<0.001	<0.001	0.656	<0.001	<0.01	<0.001	<0.05	<0.001	<0.001	<0.001	<0.001	<0.001	0.001
FR × Y	*F*	0.242	2.676	3.653	3.367	1.011	1.951	0.477	2.519	0.678	11.19	4.495	29.87	22.31	7.623	108.265
	*p*	0.785	0.073	<0.05	<0.01	0.433	0.099	0.842	<0.05	0.509	<0.001	<0.01	<0.001	<0.001	<0.001	0.001

**Table 3:** Changes in cover, height, density, Shannon–Wiener diversity index (*H*), Evenness index (*E*), Simpson index (*D*), and Richness index (*R*) of fencing grassland community under different treatment sites

Year	Sites	Cover (%)	Height (cm)	Density(individuals/m^2^)	*R*	*H*	*D*	*E*
2013	LG	22.3 ± 2.99ab	16.38 ± 0.82cd	107.6 ± 15.95ab	3.04 ± 0.13fg	2.02 ± 0.09abc	6.55 ± 0.41abc	0.91 ± 0.04cd
	FG	31.8 ± 4.21bcd	16.49 ± 1.15cd	118.4 ± 19abc	2.32 ± 0.06bc	2.2 ± 0.09abcd	9.06 ± 0.37e	0.92 ± 0.03cd
	SG	23 ± 4.13ab	15.38 ± 2.46cd	143.6 ± 22.42bcd	2.17 ± 0.1ab	2.24 ± 0.15abcde	9.15 ± 0.55e	0.96 ± 0.04d
	CG	14 ± 1.11a	4.49 ± 0.28a	226.4 ± 21.53e	3.19 ± 0.09g	1.83 ± 0.18a	7.46 ± 0.43 cd	0.81 ± 0.03abc
2014	LG	37 ± 1.87cde	18.57 ± 0.62d	141 ± 18.41bcd	3.01 ± 0.13fg	2.44 ± 0.1cde	5.82 ± 0.34ab	0.76 ± 0.04ab
	FG	41.6 ± 2.96ef	15.41 ± 0.87cd	154.6 ± 23.08bcd	2.61 ± 0.18cde	2.46 ± 0.12de	8.36 ± 0.49de	0.85 ± 0.05abcd
	SG	41 ± 3.56def	13.77 ± 0.83bc	164.4 ± 15.74cd	2.17 ± 0.12ab	2.65 ± 0.1e	9.32 ± 0.82e	0.87 ± 0.05bcd
	CG	29.4 ± 2.18bc	11.47 ± 0.47b	174.4 ± 17.98d	2.75 ± 0.12def	2.26 ± 0.13bcde	4.99 ± 0.54a	0.72 ± 0.05a
2015	LG	38.8 ± 2.71def	18.77 ± 1.06d	81 ± 4.06a	2.75 ± 0.12def	2.24 ± 0.12abcd	5.3 ± 0.41a	0.79 ± 0.04abc
	FG	55.2 ± 3.74g	24.31 ± 1.62e	121.6 ± 13.09abcd	2.46 ± 0.11bcd	2.26 ± 0.14bcde	6.99 ± 0.49bcd	0.79 ± 0.04abc
	SG	47.2 ± 3.46fg	23.73 ± 1.04e	133 ± 13.86abcd	1.88 ± 0.08a	2.48 ± 0.14de	7.14 ± 0.55bcd	0.87 ± 0.05bcd
	CG	28.4 ± 2.34bc	13.34 ± 0.9bc	177.2 ± 12.39d	1.85 ± 0.11efg	2.01 ± 0.15ab	5.83 ± 0.45ab	0.76 ± 0.05ab

Note

Values (±*SE*) are the means of five plots in one site. Different management regimes, LG: long‐term completely fencing; FG: mid‐term completely fencing; SG: mid‐term seasonal fencing; CG: free‐grazing grassland. Significant difference within the same column is indicated by different small letters (*p* < 0.05).

Different fencing regime sites (LG, FG, and SG) had significant higher (*p* < 0.05) Shannon–Weiner index, and higher Evenness index compared to CG site among 3 survey years. 11–13 years of seasonal fencing grasslands (FG and SG) had higher Shannon–Weiner index (average increase by 13.4% in FG site and 20.8% in SG site), Evenness index (average increase by 11.8% in FG site and 17.9% in SG site), and Simpson index (average increase by 33.5% in FG site and 40.1% in SG site), but lower Richness index (average decrease by 15.9% in FG site and 29.2% in SG site) compared with free‐grazing grassland (CG). SG site had highest Shannon–Weiner, Evenness, and Simpson index, while lowest Richness index in all survey years. LG and CG sites had significantly lower (*p* < 0.05) Simpson index with a decreasing amplitude range from 16.6% to 46.5%, however significantly higher (*p* < 0.05) Richness index, with an increasing amplitude range from 5.4% to 47%, than FG and SG sites. LG site had higher Evenness index (19.7% and 15.2%), Simpson index (12.5% and 23.6%), and Richness index (1.0% and 10.5%), while lower Shannon–Weiner index (17.2% and 8.6%) in 2013 than in 2014 and 2015 (Table 3).

### 3.2 Aboveground biomass and species richness of different life‐form groups

The grassland sites with different fencing (LG, FG, and SG) had significant (*p* < 0.05) higher total AGB, while there was significant (*p* < 0.05) lower total species richness than free‐grazing grassland (CG) (Table 4). The total species richness was in order of CG > LG > SG > FG, while the total AGB was in order of FG > LG > SG > CG in all survey years which decreased from 264.67 g/cm^2^ in FG to 121.52 g/cm^2^ in CG at 2013, decreased from 328.05 g/cm^2^ in FG to 223.91 g/cm^2^ in CG at 2014, and decreased from 302.61 g/cm^2^ in FG to 141.49 g/cm^2^ in CG at 2015, respectively. The AGB of annual herbs and perennial herbs in three years at three fencing sites (FG, SG, and CG) was significantly (*p* < 0.05) greater than at CG site. The AGB of shrubs at SG site was lowest in all survey years (47.36 g/cm^2^ in 2013, 73.32 g/cm^2^ in 2014, and 49.54 g/cm^2^ in 2015, respectively). The AGB of annual herbs, perennial herbs, and total plants in all sample sites was highest at 2014, however lowest at 2013. Mid‐term fencing grasslands (FG, SG) had significantly (*p* < 0.05) fewer annual herbs, perennial herbs, and total number than long‐term fencing (LG) and free‐grazing (CG) grassland. CG site had highest species richness of shrubs in all survey years. The highest annual herbs richness of different sites occurred in 2013, displayed as 3.13 in LG, 2.73 in FG, 2.87 in SG, and 4.2 in CG, respectively, while the lowest shrubs number occurred in 2013, displayed as 0.6 in LG, 0.93 in FG, 0.6 in SG, and 1.13 in CG, respectively (Table 4).

**Table 4:** Changes in aboveground biomass (AGB) and species richness (SR) within AH (annual herbs), PH (perennial herbs), *S* (shrubs), and total plants under different treatment sites

Year	Site	AGB				SR			
		AH	PH	*S*	Total	AH	PH	*S*	Total
2013	LG	23.4 ± 3.6ab	114.07 ± 11.26bcd	114.31 ± 5.13e	251.78 ± 10.05de	3.13 ± 0.32c	3.6 ± 0.31def	0.6 ± 0.13a	7.33 ± 0.27de
	FG	21.44 ± 1.83ab	144.69 ± 7.66e	108.54 ± 7.91e	274.67 ± 13.47efg	2.73 ± 0.34c	2.2 ± 0.37a	0.93 ± 0.12abc	5.87 ± 0.22abc
	SG	35.35 ± 3.48cd	104.16 ± 7.6bc	47.36 ± 6.6a	186.87 ± 11.33b	2.87 ± 0.24c	2.33 ± 0.25ab	0.6 ± 0.13a	5.8 ± 0.28ab
	CG	15.86 ± 2.02a	41.43 ± 2.63a	64.23 ± 2.82ab	121.52 ± 5.45a	4.2 ± 0.22d	3.33 ± 0.21cde	1.13 ± 0.24bc	8.67 ± 0.42f
2014	LG	50.79 ± 4.68f	128.93 ± 10.06de	112.01 ± 7.57e	291.73 ± 4.82fg	2.87 ± 0.19c	4.4 ± 0.35fg	0.8 ± 0.11ab	8.07 ± 0.32ef
	FG	46.69 ± 4.15ef	176.65 ± 8.13f	104.71 ± 8.13e	328.05 ± 17.47h	1.73 ± 0.15ab	3.07 ± 0.21bcd	1.2 ± 0.2bcd	6 ± 0.34abc
	SG	65.24 ± 5.75g	116.97 ± 5.3bcd	73.32 ± 7.06bc	255.53 ± 5.7e	2 ± 0.17ab	4 ± 0.29efg	0.87 ± 0.13abc	6.87 ± 0.4bcd
	CG	37.65 ± 1.91cde	103.16 ± 3.84bc	83.1 ± 3.6cd	223.91 ± 7.39cd	1.73 ± 0.27ab	4.73 ± 0.37g	1.67 ± 0.23d	8.13 ± 0.58ef
2015	LG	45.84 ± 3.26def	123.16 ± 7.8cd	97.48 ± 4.43de	266.48 ± 11.9ef	2.4 ± 0.21bc	3.6 ± 0.19def	0.93 ± 0.15abc	6.93 ± 0.21cd
	FG	35.86 ± 1.49cde	187.36 ± 6.04f	79.39 ± 8.42bc	302.61 ± 8.28gh	1.53 ± 0.22a	2.6 ± 0.19abc	1.2 ± 0.14bcd	5.33 ± 0.33a
	SG	52.44 ± 4.97f	98.97 ± 4.13b	49.54 ± 3.84a	200.95 ± 9.05bc	2 ± 0.22ab	3.4 ± 0.25cde	1.33 ± 0.19bcd	6.73 ± 0.38bcd
	CG	27.88 ± 2.77bc	60.8 ± 2.93a	52.81 ± 2.39a	141.49 ± 3.31a	1.87 ± 0.26ab	4.47 ± 0.13g	1.4 ± 0.13cd	7.73 ± 0.27def

Note

Values (±*SE*) are means of five plots in one site. Different management regimes, LG: long‐term completely fencing; FG: mid‐term completely fencing; SG: mid‐term seasonal fencing; CG: free‐grazing grassland. Significant difference within the same life‐form group is indicated by different small letters.

### 3.3 Soil physicochemical properties

Compared with grazing grassland (CG), the exclosure grasslands (LG, FG, and SG) all significantly (*p* < 0.05) increased soil TN, TP, and SOM, while significantly (*p* < 0.05) decreased soil BD, SWC, and pH in three years (Table 5). The BD and pH in three years were all in order of CG > SG > LG > FG. SWC in LG, FG, and SG sites decreased by 48%, 51%, and 51.4% in 2013, 24.9%, 28.9%, and 26% in 2014, and 7.8%, 10.9%, and 10.4% in 2015 than CG site, respectively. Soil TN in three years was all in order of FG > SG > LG > CG, and ranging from 89.2 mg/kg in CG to 108.6 mg/kg in FG at 2013, ranging from 81.7 mg/kg in CG to 112.7 mg/kg in FG at 2014, and ranging from 78.1 mg/kg in CG to 115.9 mg/kg in FG at 2015, respectively. Soil TP and SOM showed similar trend as TN. SWC, TN, TP, and SOM had significant difference (*p* < 0.05) among all sites in all survey years. However, soil BD between LG and FG sites in 2014, and pH between LG and FG sites in all years had no significant difference (*p* > 0.05) (Table 5).

**Table 5:** Changes in soil bulk density (BD), soil water content (SWC), total nitrogen (TN), total phosphorus (TP), soil organic matter (SOM), and pH

Time	Sites	BD (g/cm^3^)	SWC (%)	TN (mg/kg)	TP (mg/kg)	SOM (g/kg)	pH
2013	LG	1.89 ± 0.059a	108.58 ± 0.64f	190.27 ± 0.85e	4.54 ± 0.1c	8.01 ± 0.05ab	8.01 ± 0.05ab
	FG	2.73 ± 0.068c	102.47 ± 0.87de	226 ± 1.73h	5.17 ± 0.08ef	7.91 ± 0.04a	7.91 ± 0.04a
	SG	3.36 ± 0.036e	99.13 ± 0.95cd	204.14 ± 1.85f	4.83 ± 0.09d	8.26 ± 0.07c	8.26 ± 0.07c
	CG	3.64 ± 0.092f	89.19 ± 0.61b	146.58 ± 5.16c	4.29 ± 0.12c	8.51 ± 0.06d	8.51 ± 0.06d
2014	LG	2.02 ± 0.054ab	97.46 ± 0.89c	186.38 ± 0.78de	4.42 ± 0.09c	8.09 ± 0.06b	8.09 ± 0.06b
	FG	2.93 ± 0.059d	112.73 ± 1.17g	230.33 ± 1.35hi	5.37 ± 0.11fg	7.96 ± 0.05ab	7.96 ± 0.05ab
	SG	3.68 ± 0.084f	103.88 ± 0.73e	209.2 ± 0.96 fg	4.94 ± 0.08de	8.29 ± 0.05c	8.29 ± 0.05c
	CG	4.13 ± 0.071g	81.72 ± 0.72a	126.14 ± 2.47b	3.86 ± 0.13b	8.64 ± 0.04e	8.64 ± 0.04e
2015	LG	2.17 ± 0.026b	96.35 ± 173c	183.04 ± 2.83d	4.33 ± 0.08c	8.1 ± 0.04b	8.1 ± 0.04b
	FG	3.31 ± 0.04e	115.92 ± 2.59g	234.28 ± 1.9i	5.48 ± 0.04g	8.04 ± 0.03ab	8.04 ± 0.03ab
	SG	4.01 ± 0.028g	106 ± 2.06ef	214.43 ± 2.56g	5.01 ± 0.05de	8.33 ± 0.04c	8.33 ± 0.04c
	CG	4.48 ± 0.024h	78.13 ± 1.05a	111.41 ± 1.22a	3.48 ± 0.04a	8.73 ± 0.03e	8.73 ± 0.03e

Note

Values (±*SE*) are means of five samples in one site. Different management regimes, LG: long‐term completely fencing; FG: mid‐term completely fencing; SG: mid‐term seasonal fencing; CG: free‐grazing grassland. Significant difference within the same column is indicated by different small letters.

### 3.4 Relationship between community properties and soil physicochemical properties

As shown in Table 6, the AGB was significantly positive related to TN (*p* = 0.017), TP (*p* = 0.016), and SOM (*p* = 0.036), while negative related with BD (*p* = 0.001), SWC (*p* = 0.036), and pH (*p* = 0.002). Plant cover and height were significantly correlated with soil TN and TP (*p* < 0.05). Simpson index was significantly related (*p* < 0.05) with TN, TP, and SOM. There had no significant (*p* > 0.05) relationships between SWC and plant cover, and height, while had significant (*p* < 0.05) relationship between SWC and plant density (Table 6).

**Table 6:** Pearson's correlation coefficients between community properties and soil physicochemical properties

	BD	SWC	TN	TP	SOM	pH
AGB	−0.822**	−0.607*	0.671*	0.673*	0.608*	−0.804**
R	0.093	−0.325	−0.542	−0.579*	−0.569	0.150
H	−0.275	−0.009	0.502	0.546	0.489	−0.293
D	−0.002	0.056	0.602*	0.620*	0.669*	−0.357
E	−0.140	−0.231	0.544	0.600*	0.557	−0.480
Cover	−0.562	0.001	0.611*	0.585*	0.562	−0.368
Height	−0.684*	−0.265	0.610*	0.620*	0.513	−0.518
Density	0.745**	0.643*	−0.477	−0.530	−0.356	0.689*

Note

AGB: aboveground biomass; BD: soil bulk density; *D*: Simpson index; *E*: Evenness index; *H*: Shannon–Wiener index; *R*: Richness index; SOM: soil organic matter; SWC: soil water content; TN: total nitrogen; TP: total phosphorus.

Significant difference is indicated by symbols, ^*^ *p* < 0.05, ^**^ *p* < 0.01.

## 4 DISCUSSION

The restoration of degraded grasslands ecosystem after fencing is a complex and long‐term ecological process (Zhu et al., 2016). Previous studies have shown that removing grazing could reduce nutrient and energy cycling from soil–vegetation ecosystem to livestock, and this has significant remarkable impact on vegetation index, diversity index, aboveground biomass, and soil nutrients (Harris, Moretto, Distel, Boutton, & Roberto, 2007; Liu et al., 2015; Wu et al., 2009; Zeng, Liu, Xiao, & Huang, 2017; Zhu et al., 2016). Our results indicated that 11–13 years (moderate time) of fencing would allow vegetation and soil nutrients to recover, while 22–24 years of exclosure might cause a redegradation trend of some community and soil properties. This is consistent with studies in other grasslands (Angassa & Oba, 2010; Liu & Zhang, 2018; Shang et al., 2010; Su et al., 2015; Yan & Tang, 2007). The 11–13 years of seasonal fencing grassland had greater plant density, species richness, biodiversity, and soil physical properties than contemporaneous fencing grassland.

Because there is a lack of disturbance from human and livestock, such as grazing, mowing, and farming (Medinaroldán, Paz‐Ferreiro, & Bardgett, 2012), fencing could improve ecological environment of the vegetation for a short time (Catford et al., 2012; Rooyen et al., 2014; Shang et al., 2013). In the early days of exclosure, empty community niches, due to livestock consumption, are gradually taken up by restoring vegetation (Zeng et al., 2017; Zou et al., 2016). The proportion of some palatable perennial grasses increased due to abundant nutrients and water resources (Batoyun, Shinoda, Cheng, & Purevdorj, 2016), but annual herbs trended to decrease (Wu et al., 2009; Zheng, Cao, & Wang, 2005). The colonization capacity of different plants influences the effects of vegetation restoration (Shang et al., 2013). The changing of community composition may induce a further impact on ecosystem (Deng et al., 2014; Mekuria & Aynekulu, 2013). Our results indicated that 11–13 years of exclosure had a positive effect on plant cover, height, total AGB, Shannon–Weiner index, and Evenness index, while a negative effect on plant density, total species richness, and Richness index. The restoration of vegetation also effectively prevents erosion by rainfall and improves nutrients retention (Zhang, Yang, & Zepp, 2004). There is also an increase in litter input which improves soil nutrient content (Li, Zhang, et al., 2017; Li, Cao, et al., 2017; Medinaroldán et al., 2012; Mekuria & Aynekulu, 2013). The improvement of soil structure and the environment, in turn, promote the growth and development of vegetation (Deng et al., 2014; Wu et al., 2010; Zhu et al., 2016).

However, as fencing time increases, the competition (intra‐ and interspecific) increased because of the limited natural resources such as light, water, and nutrient (Huston, 1994; Oba et al., 2001; Wal et al., 2004). Some research notes that the restoration process of degraded ecosystem is accompanied with community succession (Deng et al., 2014; Shang et al., 2010; Wu et al., 2009). In restoring grasslands, some tall and strong germinating plants with stronger water and nutrient use, especially *Kobresia *groups in the study area (Liu & Zhang, 2018), are gradually demonstrating their competitive advantage in the process of community succession after long‐term fencing, which inhibit the growth of annual groups in herbaceous layer (Deng et al., 2014). After enclosed for a certain time, the litter accumulation combines with the formation and development of soil crust impedes infiltration of precipitation (Aubert et al., 2011), which deteriorates soil moisture (Oba et al., 2001; Read, Duncan, Vesk, & Elith, 2011; Yang, Chu, Chen, Wang, & Bai, 2014). The decrease in soil moisture directly affects the activity of the soil microbial communities and limits their capacity to decompose certain compounds, leading to the decrease in soil nutrients (Hueso, García, & Hernández, 2012). In addition, lower soil moisture restrains the growing development of seedlings and the decomposition of litters and dead roots (Čatský, 2001; Yan, Tang, Xin, & Wang, 2009), and reduces the circulation velocity of energy in plant–soil ecosystem (Harris et al., 2007). The increase of annual grasses after long‐term fencing results in less root which also has negative effects on the accumulation of soil nutrients (Čatský, 2001). As a consequence, after long‐term fencing, grasslands may experience have a degradation trend in plant and soil physicochemical properties (Angassa & Oba, 2010; Su et al., 2015), characterized by lower plant cover, density, total AGB, biodiversity, soil TN, TP, and SOM in LG site than in FG site.

Grazing, as one of the major traction of vegetation succession in northwest China, has some passive effects on germination, growth, development, mortality, and propagation of plant species, and in turn leads to shifts in community quantity characteristics and diversities (Mekuria & Aynekulu, 2013; Wassie, Sterck, Teketay, & Bongers, 2009). Studies have shown that different management regimes had dissimilar influences on grassland ecosystem (Li, Zhang, et al., 2017; Li, Cao, et al., 2017; Mekuria & Aynekulu, 2013). The selective feeding and patchy defecation of livestock increase soil nutrients by accelerating circulation flow of material energy, which accelerates the restoration of degraded soil and causes variation in community composition (Harris et al., 2007; Li, Cao, et al., 2017; Sheppard, Hodge, Paynter, & Rees, 2002). On the one hand, livestock are more likely to mistaken eat the seeds of palatable perennial herbs when ingest leaves. And then, these seeds excrete with feces which aggravates the distribution of perennial herbs (Bertiller & Ares, 2011). Conversely, this behavior provides a better living environment for seed germination, increases soil fertility, and enhances environmental heterogeneity, which provides favorable conditions for the invasion of alien species (Keeley, Lubin, & Fotheringham, 2003; Zuo et al., 2008). The activities of livestock, such as grazing and trampling, not only directly impact aboveground productivity of dominant species, weaken their competitive advantage, and reduce the capacity to utilize resources, but also reduce the barrier effect of surface crust on seeds and promote the growth of lower perennial and annual herbs (Chartier et al., 2011; Gómez et al., 2012; Inderjit, 2005; Miao et al., 2015). These factors increase the invasiveness of community and lead to increase in species richness and decrease in biodiversity (Brooker et al., 2008; Chambers et al., 2014). Therefore, according to “intermediate disturbance hypothesis,” appropriate human interference, such as seasonal grazing, should be taken to maintain plant cover and biodiversity on fencing grasslands after excluding the interference of human and herbivorous livestock for appropriate time.

## 5 CONCLUSIONS

In this study, moderate time fencing (11–13 years) could improve some plant properties and soil nutrients, while 22–24 years of long‐term fencing might cause a decreasing trend of those vegetation and soil indexes which indicated the redegradation of grassland ecosystem. Additionally, seasonal fencing grassland (11–13 years) was better to maintain biodiversity. All these results indicated that different fencing regimes are all effective to recover the degraded desert grasslands in Mu Us desert. Our results lead us to recommend that winter grazing and summer fencing could be used as a beneficial management strategy.

## CONFLICTS OF INTEREST

None declared.

## AUTHOR CONTRIBUTIONS

Jiankang Liu and Zhen Bian involved in data curation. Jiankang Liu involved in formal analysis. Kebin Zhang acquired funding. Jiankang Liu, Zhen Bian, and Kebin Zhang involved in methodology. Bilal Ahmad and Alamgir Khan involved in visualization. Jiankang Liu involved in writing of the original draft. Kebin Zhang involved in writing, reviewing, and editing of the manuscript.

## Data Availability

I agree to deposit my data to a public repository. All data input files: figshare https://doi.org/10.6084/m9.figshare.7053701.

## References

[ece34958-bib-0001] Akiyama, T. , & Kawamura, K. (2007). Grassland degradation in China: Methods of monitoring, management and restoration. Grassland Science, 53(1), 1–17. 10.1111/j.1744-697X.2007.00073.x

[ece34958-bib-0002] Angassa, A. , & Oba, G. (2010). Effects of grazing pressure, age of enclosures and seasonality on bush cover dynamics and vegetation composition in southern Ethiopia. Journal of Arid Environments, 74(1), 111–120. 10.1016/j.jaridenv.2009.07.015

[ece34958-bib-0003] Aubert, M. , Baghdadi, N. , Zribi, M. , Douaoui, A. , Loumagne, C. , Baup, F. , … Garrigues, S. (2011). Analysis of terrasar‐x data sensitivity to bare soil moisture, roughness, composition and soil crust. Remote Sensing of Environment, 115(8), 1801–1810. 10.1016/j.rse.2011.02.021

[ece34958-bib-0004] Batoyun, T. , Shinoda, M. , Cheng, Y. , & Purevdorj, Y. (2016). Effects of grazing and precipitation variability on vegetation dynamics in a mongolian dry steppe. Journal of Plant Ecology, 9(1–5), 508–519. 10.1093/jpe/rtv083

[ece34958-bib-0005] Bertiller, M. B. , & Ares, J. O. (2011). Does sheep selectivity along grazing paths negatively affect biological crusts and soil seed banks in arid shrublands? A case study in the Patagonian Monte. Argentina. Journal of Environmental Management, 92(8), 2091–2096. 10.1016/j.jenvman.2011.03.027 21511391

[ece34958-bib-0006] Bremner, J. M. (1996). Nitrogen‐total In Sparks D. L. (Ed.), Methods of soil analysis, Part 3. SSSA Book Series. Madison, WI: America Society of Agronomy.

[ece34958-bib-0007] Brooker, R. W. , Maestre, F. T. , Callaway, R. M. , Lortie, C. L. , Cavieres, L. A. , Kunstler, G. , … Anthelme, F. (2008). Facilitation in plant communities: The past, the present, and the future. Journal of Ecology, 96(1), 18–34. 10.1111/j.1365-2745.2007.01295.x

[ece34958-bib-0008] Catford, J. A. , Daehler, C. C. , Murphy, H. T. , Sheppard, A. W. , Hardesty, B. D. , Westcott, D. A. , … Hulme, P. E. (2012). The intermediate disturbance hypothesis and plant invasions: Implications for species richness and management. Perspectives in Plant Ecology Evolution & Systematics, 14(3), 231–241. 10.1016/j.ppees.2011.12.002

[ece34958-bib-0009] Čatský, J. (2001). Follett, r.f. kimble, j.m. lal, r. (ed.): the potential of u.s. grazing lands to sequester carbon and mitigate the greenhouse effects. Photosynthetica, 39(2), 182 10.1023/A:1013769712551

[ece34958-bib-0010] Chambers, J. C. , Bradley, B. A. , Brown, C. S. , D'Antonio, C. , Germino, M. J. , Grace, J. B. , … Pyke, D. A. (2014). Resilience to stress and disturbance, and resistance to bromus tectorum l. invasion in cold desert shrublands of western North America. Ecosystems, 17(2), 360–375. 10.1007/s10021-013-9725-5

[ece34958-bib-0011] Chartier, M. P. , Rostagno, C. M. , & Pazos, G. E. (2011). Effects of soil degradation on infiltration rates in grazed semiarid rangelands of northeastern Patagonia, Argentina. Journal of Arid Environments, 75(7), 656–661. 10.1016/j.jaridenv.2011.02.007

[ece34958-bib-0012] Connell, J. H. (1979). Intermediate‐disturbance hypothesis. Science, 204(4399), 1344–1345. 10.1126/science.204.4399.1344 17813175

[ece34958-bib-0013] Deng, L. , Zhang, Z. , & Shangguan, Z. (2014). Long‐term fencing effects on plant diversity and soil properties in China. Soil and Tillage Research, 137, 7–15. 10.1016/j.still.2013.11.002

[ece34958-bib-0015] Gao, Q. Z. , Li, Y. , Wan, Y. F. , Jiangcun, W. Z. , Qin, X. B. , & Wang, B. S. (2009). Significant achievements in protection and restoration of alpine grassland ecosystem in Northern Tibet, China. Restoration Ecology, 17(3), 320–323. 10.1111/j.1526-100X.2009.00527.x

[ece34958-bib-0016] Gómez, D. A. , Aranibar, J. N. , Tabeni, S. , Villagra, P. E. , Garibotti, I. A. , & Atencio, A. (2012). Biological soil crust recovery after long‐term grazing exclusion in the monte desert (Argentina). Changes in coverage, spatial distribution, and soil nitrogen. Acta Oecologica, 38, 33–40. 10.1016/j.actao.2011.09.001

[ece34958-bib-0017] Harris, W. N. , Moretto, A. S. , Distel, R. A. , Boutton, T. W. , & Roberto, M. B. (2007). Fire and grazing in grasslands of the argentine caldenal: Effects on plant and soil carbon and nitrogen. Acta Oecologica, 32(2) , 207–214. 10.1016/j.actao.2007.05.001

[ece34958-bib-0018] He, Y. H. , Zhao, H. L. , Liu, X. P. , Zhao, X. Y. , & Li, Y. Q. (2008). Soil physical and chemical characteristics of sandy meadow in natural restoration process. Journal of Soil & Water Conservation, 22(2), 159–162. 10.3321/j.issn:1009-2242.2008.02.036

[ece34958-bib-0019] Hobbs, R. J. , & Huenneke, L. F. (1992). Disturbance, diversity, and invasion: Implications for conservation. Conservation Biology, 6(3), 324–337. 10.1046/j.1523-1739.1992.06030324.x

[ece34958-bib-0020] Hueso, S. , García, C. , & Hernández, T. (2012). Severe drought conditions modify the microbial community structure, size and activity in amended and unamended soils. Soil Biology & Biochemistry, 50, 167–173. 10.1016/j.soilbio.2012.03.026

[ece34958-bib-0021] Huston, M. A. (1994). Biological diversity: The coexistence of species on changing landscapes. Cambridge, UK: Cambridge University Press.

[ece34958-bib-0022] Huxman, T. E. , Smith, M. D. , Fay, P. A. , Knapp, A. K. , Shaw, M. R. , Loik, M. E. , … Williams, D. G. (2004). Convergence across biomes to a common rain‐use efficiency. Nature, 429(6992), 651–654. 10.1038/nature02561 15190350

[ece34958-bib-0023] Inderjit (2005). Soil microorganisms: An important determinant of allelopathic activity. Plant & Soil, 274(1–2), 227–236. 10.1007/s11104.004.0159.x

[ece34958-bib-0024] Keeley, J. E. , Lubin, D. , & Fotheringham, C. J. (2003). Fire and grazing impacts on plant diversity and alien plant invasions in the southern Sierra Nevada. Ecological Applications, 13(5), 1355–1374. 10.1890/02-5002

[ece34958-bib-0025] Lal, R. (2004). Soil carbon sequestration impacts on global climate change and food security. Science, 304(5677), 1623–1627. 10.1126/science.1097396 15192216

[ece34958-bib-0026] Li, J. , Zhang, C. , Yang, Z. , Guo, H. , Zhou, X. , & Du, G. (2017). Grazing and fertilization influence plant species richness via direct and indirect pathways in an alpine meadow of the eastern Tibetan plateau. Grass and Forage Science, 72(2), 343–354. 10.1111/gfs.12232

[ece34958-bib-0027] Li, W. , Cao, W. , Wang, J. , Li, X. , Xu, C. , & Shi, S. (2017). Effects of grazing regime on vegetation structure, productivity, soil quality, carbon and nitrogen storage of alpine meadow on the Qinghai‐Tibetan Plateau. Ecological Engineering, 98, 123–133. 10.1016/j.ecoleng.2016.10.026

[ece34958-bib-0028] Liu, J. , & Zhang, K. (2018). Spatial pattern and population structure of *Artemisia ordosica* shrub in a desert grassland under enclosure, Northwest China. International Journal of Environmental Research and Public Health, 15, 946 10.3390/ijerph15050946 PMC5981985 29747420

[ece34958-bib-0029] Liu, W. , Xie, Y. , Dai, H. , & Rao, D. (2008). Study on community growth of Stipa bungeana population under different grazing intensities in desert steppe. Journal of Agricultural Sciences, 2, 50–53. 10.3969/j.issn.1673-0747.2008.02.014

[ece34958-bib-0030] Liu, X. , Zhang, K. , Wang, L. , & Yang, L. (2015). Studies on plant community complexity in fenced region of Ningxia, Northern China. Journal of Beijing Forestry University, 37(2), 48–54. 10.13332/j.cnki.jbfu.2015.02.010

[ece34958-bib-0031] Ma, W. , Ding, K. , & Li, Z. (2016). Comparison of soil carbon and nitrogen stocks at grazing‐excluded and yak grazed alpine meadow sites in Qinghai‐Tibetan Plateau, China. Ecological Engineering, 87, 203–211. 10.1016/j.ecoleng.2015.11.040

[ece34958-bib-0032] Medinaroldán, E. , Paz‐Ferreiro, J. , & Bardgett, R. D. (2012). Grazing exclusion affects soil and plant communities, but has no impact on soil carbon storage in an upland grassland. Agriculture, Ecosystems & Environment, 149, 118–123. 10.1016/j.agee.2011.12.012

[ece34958-bib-0033] Mekuria, W. , & Aynekulu, E. (2013). Exclosure land management for restoration of the soils in degraded communal grazing lands in northern Ethiopia. Land Degradation & Development, 24(6), 528–538. 10.1002/ldr.1146

[ece34958-bib-0034] Miao, F. , Guo, Z. , Xue, R. , Wang, X. , & Shen, Y. (2015). Effects of grazing and precipitation on herbage biomass, herbage nutritive value, and yak performance in an Alpine Meadow on the Qinghai‐Tibetan Plateau. PLoS ONE, 10(6), e0127275 10.1371/journal.pone.0127275 26039322 PMC4454548

[ece34958-bib-0035] Nedessa, B. , Ali, J. , & Nyborg, I. (2005). Exploring ecological and socio‐economic issues for the improvement of area enclosure management: a case study from Ethiopia. Drylands Coordination Group Report, 12(1), 95–107. 10.1017/S1461145708009140

[ece34958-bib-0036] Nelson, D. W. , & Sommers, L. E. (1982). Total carbon, organic carbon and organic matter In Klute A. (Ed.), Methods of soil analysis. Part 3. Chemical methods. Madison, WI: Soil Science of America and American Society of Agronomy.

[ece34958-bib-0037] Oba, G. , Vetaas, O. R. , & Stenseth, N. C. (2001). Relationships between biomass and plant species richness in arid‐zone grazing lands. Journal of Applied Ecology, 38(4), 836–845. 10.1046/j.1365-2664.2001.00638.x

[ece34958-bib-0038] Parkinson, J. A. , & Allen, S. E. (1975). A wet oxidation procedure suitable for the determination of nitrogen and mineral nutrients in biological material. Communications in Soil Science and Plant Analysis, 6(1), 1–11. 10.1080/00103627509366539

[ece34958-bib-0039] Read, C. F. , Duncan, D. H. , Vesk, P. A. , & Elith, J. (2011). Surprisingly fast recovery of biological soil crusts following livestock removal in southern Australia. Journal of Vegetation Science, 22(5), 905–916. 10.1111/j.1654-1103.2011.01296.x

[ece34958-bib-0040] Ren, X. , Jia, Z. , Wan, S. , Han, Q. , & Chen, X. (2011). The long‐term effects of alfalfa on soil water content in the Loess Plateau of northwest China. African Journal of Biotechnology, 10(21), 4420–4427. 10.5897/AJB10.2678

[ece34958-bib-0041] Rooyen, M. W. V. , Roux, A. L. , Geldenhuys, C. , Rooyen, N. V. , & Merwe, H. V. D. (2014). Long‐term vegetation dynamics (40 yr) in the succulent karoo, South Africa: Effects of rainfall and grazing. Applied Vegetation Science, 18(2), 311–322. 10.1111/avsc.12150

[ece34958-bib-0042] Schmiede, R. , Donath, T. W. , & Otte, A. (2009). Seed bank development after the restoration of alluvial grassland via transfer of seed‐containing plant material. Biological Conservation, 142(2), 404–413. 10.1016/j.biocon.2008.11.001

[ece34958-bib-0043] Shang, Z. H. , Ma, Y. S. , Long, R. J. , & Ding, L. M. (2010). Effect of fencing, artificial seeding and abandonment on vegetation composition and dynamics of ‘black soil land’ in the headwaters of the yangtze and the yellow rivers of the qinghai‐tibetan plateau. Land Degradation & Development, 19(5), 554–563. 10.1002/ldr.861

[ece34958-bib-0044] Shang, Z. , Deng, B. , Ding, L. , Ren, G. , Xin, G. , Liu, Z. , … Long, R. (2013). The effects of three years of fencing enclosure on soil seed banks and the relationship with above‐ground vegetation of degraded alpine grasslands of the tibetan plateau. Plant and Soil, 364(1–2), 229–244. 10.1007/s11104-012-1362-9

[ece34958-bib-0045] Sheppard, A. W. , Hodge, P. , Paynter, Q. , & Rees, M. (2002). Factors affecting invasion and persistence of broom cytisus scoparius in Australia. Journal of Applied Ecology, 39(5), 721–734. 10.1046/j.1365-2664.2002.00750.x

[ece34958-bib-0046] Socher, S. A. , Prati, D. , Boch, S. , Müller, J. , & Fischer, M. (2012). Direct and productivity‐mediated indirect effects of fertilization, mowing and grazing on grassland species richness. Journal of Ecology, 100(6), 1391–1399. 10.1111/j.1365-2745.2012.02020.x

[ece34958-bib-0047] Su, H. , Liu, W. , Xu, H. , Wang, Z. , & Li, Y. (2015). Long‐term livestock exclusion facilitates native woody plant encroachment in a sandy semiarid rangeland. Ecology and Evolution, 5(12), 2445–2456. 10.1002/ece3.1531 26120433 PMC4475376

[ece34958-bib-0048] Ulanowicz, R. E. (2001). Information theory in ecology. Computers & Chemistry, 25(4), 393–399. 10.1016/S0097-8485(01)00073-0 11459353

[ece34958-bib-0049] Wal, R. V. D. , Bardgett, R. D. , Harrison, K. A. , & Stien, A. (2004). Vertebrate herbivores and ecosystem control: Cascading effects of faeces on tundra ecosystems. Ecography, 27(2), 242–252. 10.1111/j.0906-7590.2004.03688.x

[ece34958-bib-0050] Wassie, A. , Sterck, F. J. , Teketay, D. , & Bongers, F. (2009). Effects of livestock exclusion on tree regeneration in church forests of Ethiopia. Forest Ecology and Management, 257, 765–772. 10.1016/j.foreco.2008.07.032

[ece34958-bib-0051] Wu, G. , Du, G. , Liu, Z. , & Thirgood, S. (2009). Effect of fencing and grazing on a Kobresia‐dominated meadow in the Qinghai‐Tibetan plateau. Plant and Soil, 319(1–2), 115–126. 10.1016/S0145-305X(00)00063-X

[ece34958-bib-0052] Wu, G. L. , Liu, Z. H. , Zhang, L. , Chen, J. M. , & Hu, T. M. (2010). Long‐term fencing improved soil properties and soil organic carbon storage in an alpine swamp meadow of western China. Plant and Soil, 332(1–2), 331–337. 10.1007/s11104-010-0299-0

[ece34958-bib-0053] Wu, J. G. , & Loucks, O. (1992). Grasslands and grassland sciences in Northern China. U. S. National Research Council edition. Washington, DC: National Academy Press.

[ece34958-bib-0054] Yan, Y. C. , & Tang, H. P. (2007). Effects of enclosure on Typical Steppe community properties in Inner Mongolia. Acta Botanica Boreali‐Occidentalia Sinica, 27(6), 1225–1232. 10.3321/j.issn:1000-4025.2007.06.026

[ece34958-bib-0055] Yan, Y. C. , Tang, H. P. , Xin, X. P. , & Wang, X. (2009). Advances in research on effects of exclosure on grasslands. ACTA Ecology Sinica, 29(9), 5039–5046. 1000‐0933(2009)09‐5039‐08

[ece34958-bib-0056] Yang, J. , Chu, P. F. , Chen, D. M. , Wang, M. J. , & Bai, Y. F. (2014). Mechanisms underlying the impacts of grazing on plant α, β and γ diversity in a typical steppe of the Inner Mongolia grassland. Chinese Journal of Plant Ecology, 2, 188–200. 10.3724/SP.J.1258.2014.00017

[ece34958-bib-0058] Zeng, Q. , Liu, Y. , Xiao, L. , & Huang, Y. (2017). How fencing affects the soil quality and plant biomass in the grassland of the loess plateau. . International Journal of Environmental Research and Public Health, 14(10), 1117 10.3390/ijerph14101117 PMC5664618 28946681

[ece34958-bib-0059] Zhang, B. , Yang, Y. S. , & Zepp, H. (2004). Effect of vegetation restoration on soil and water erosion and nutrient losses of a severely eroded clayey plinthudult in Southeastern China. Catena, 57(1), 77–90. 10.1016/j.catena.2003.07.001

[ece34958-bib-0060] Zheng, C. , Cao, Z. , & Wang, X. (2005). Effects of enclosure on vegetation's recovery indesertified grassland in Hulunbeir. Science of Soil and Water Conservation, 3, 78–81. 10.3969/j.issn.1672-3007.2005.03.016

[ece34958-bib-0061] Zhu, G. Y. , Deng, L. , Zhang, X. B. , & Shangguan, Z. P. (2016). Effects of grazing exclusion on plant community and soil physicochemical properties in a desert steppe on the Loess Plateau, China. Ecological Engineering, 90, 372–381. 10.1016/j.ecoleng.2016.02.001

[ece34958-bib-0062] Zou, J. , Luo, C. , Xu, X. , Zhao, N. , Zhao, L. , & Zhao, X. (2016). Relationship of plant diversity with litter and soil available nitrogen in an alpine meadow under a 9‐year grazing exclusion. Ecological Research, 31(6), 841–851. 10.1007/s11284-016-1394-3

[ece34958-bib-0063] Zuo, L. , Zhang, Z. , Zhao, X. , Xiao, W. , Wu, W. , Ling, Y. , & Liu, F. (2014). Multitemporal analysis of cropland transition in a climate‐sensitive area: A case study of the arid and semiarid region of northwest china. Regional Environmental Change, 14(1), 75–89. 10.1007/s10113-013-0435-5

[ece34958-bib-0064] Zuo, X. , Zhao, H. , Zhao, X. , Zhang, T. , Guo, Y. , Wang, S. , … Sam, D. (2008). Spatial pattern and heterogeneity of soil properties in sand dunes under grazing and restoration in Horqin Sandy Land, Northern China. Soil and Tillage Research, 99(2), 202–212. 10.1016/j.still.2008.02.008

